# Borderline lepromatous leprosy with lymph node infiltration: Dermatology helps to clarify challenging diagnoses^☆^

**DOI:** 10.1016/j.abd.2024.10.004

**Published:** 2025-04-03

**Authors:** Victor Henrique Murback dos Reis, Anna Carolina Miola, Cesar Augusto Zago Ferreira, Simone de Pádua Milagres, Joel Carlos Lastória, Juliano Vilaverde Schmitt

**Affiliations:** Department of Infectology, Dermatology, Imaging Diagnosis and Radiotherapy, Faculty of Medicine, Universidade Estadual Paulista, Botucatu, SP, Brazil

*Dear Editor,*

Eliminating the occurrence of leprosy (L) is a global goal of the WHO. For this purpose, combating the disease in Brazil is of utmost importance, since the country accounts for 13% of the absolute number of new cases of the disease worldwide.^1^

Early identification of possible cases of L in the community, whether by general practitioners, medical doctors of different specialties, or dermatologists, is essential to reduce the spread of the disease and combat sequelae related to late diagnosis. Therefore, it is essential to invest in diagnostic training for the medical community, nurses and community health agents.^2^

It is necessary, however, to keep in mind that many cases do not follow the classic disease presentation, making the diagnosis difficult, which also makes it necessary to get to know disease presentations in which the manifestations go beyond the involvement of cutaneous and neurological sites.

The present report describes a 48-year-old male patient with testicular pain radiating to the inguinal region, chills, insomnia, night sweats and spontaneous weight loss of 9 kg in two months. On physical examination, the patient had bilateral inguinal lymphadenopathy, with mobile lymph nodes, with a parenchymal appearance, measuring 3 cm in diameter, without involvement of other lymph node chains. On ultrasound examination, the enlarged inguinal lymph nodes presented a reactive architecture. There was also splenomegaly on the abdominal ultrasound. The blood count showed lymphopenia.

The internal medicine team hypothesized non-Hodgkin's lymphoma as the diagnosis, and a biopsy of one of the lymph nodes was performed. Histopathology showed lymph node stroma disorganization with a marked number of histiocytes. On Fite-Faraco staining, numerous acid-fast bacilli organized in clusters were observed, confirming the diagnosis of lymph node involvement by L (Fig. 1).

**Fig. 1:**
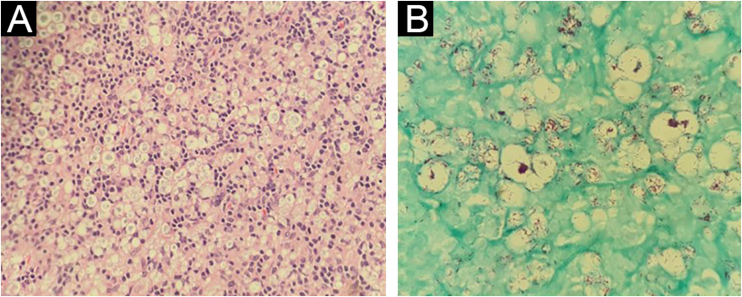
Inguinal lymph node histopathology – (A) Presence of a diffuse histiocytic infiltrate, lymph node stroma disorganization and presence of typical lymphocytes (Hematoxylin & eosin, ×40); (B) Presence of numerous acid-fast bacilli, organized in clusters within xanthomatous histiocytes (Fite-Faraco, ×100).

During the consultation with Dermatology, a hypochromic patch on the left infrascapular region was observed on physical examination, which was anesthetic on esthesiometry examination (Fig. 2, Fig. 3). Additionally, the entire perilesional area showed apparent diffuse cutaneous infiltration. Painless thickening of the ulnar nerves bilaterally and discrete infiltration of the earlobes were also observed.

**Fig. 2:**
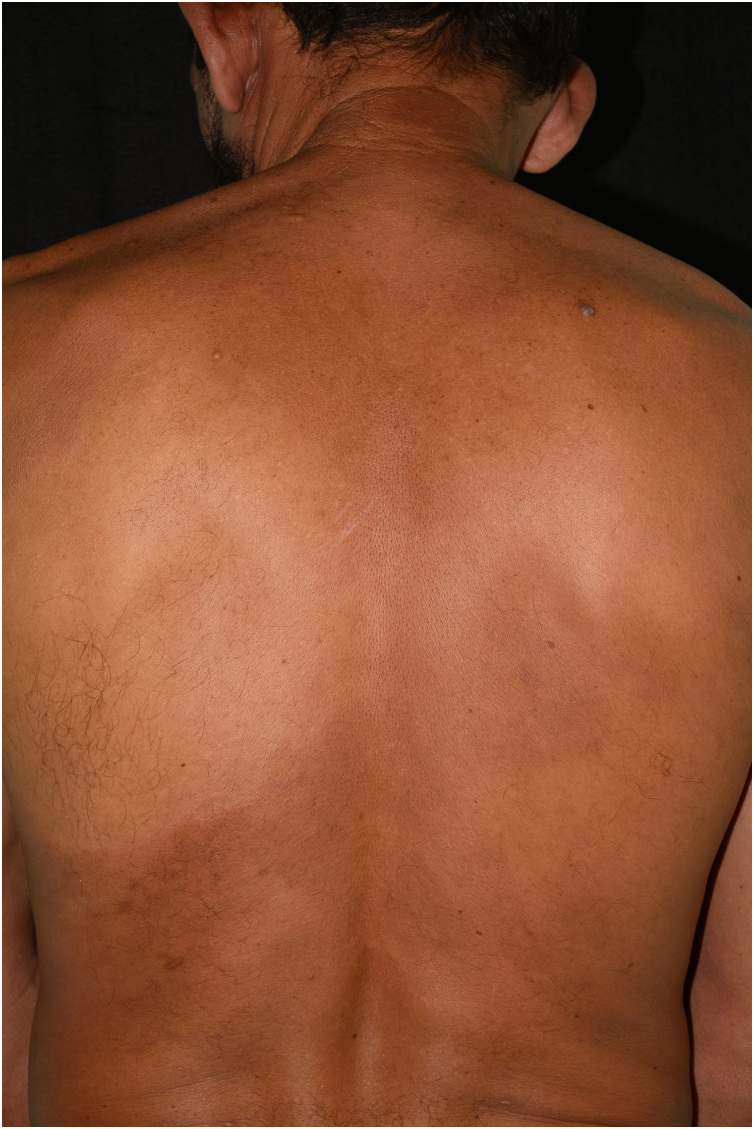
Hypochromic spot affecting the scapular and infrascapular region, extending to the left costal region, anesthetic on esthesiometry examination. On the periphery of the lesion, a discreet, poorly delimited erythematous-brownish area is observed.

**Fig. 3:**
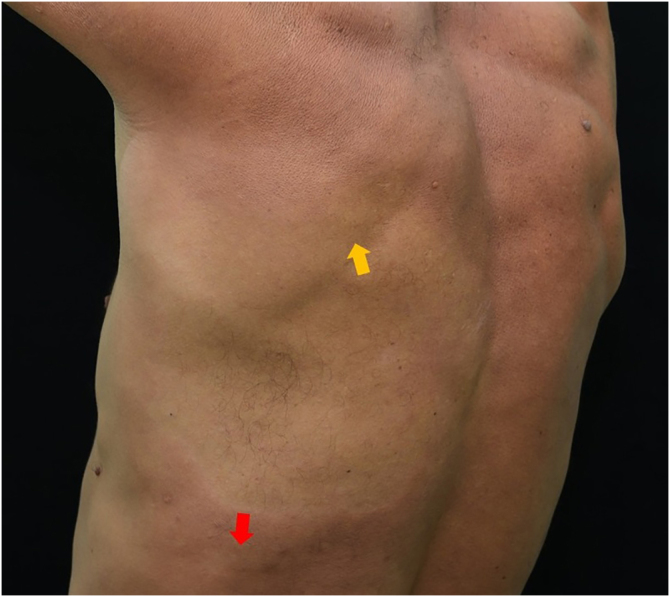
Hypochromic spot affecting the scapular and infrascapular region, extending to the anesthetic left costal region. The yellow arrow points to the biopsied area of suspected leprosy. The red arrow points to an area of discrete peripheral infiltration, which was also biopsied.

Biopsies were performed in the hypochromic infrascapular lesion, considered a suspicious lesion on physical examination, and in the perilesional region with discrete infiltration. Histopathology of the hypochromic area showed a discrete perivascular inflammatory infiltrate, negative for bacilli on Fite-Faraco staining. The histopathological findings of the infiltrated perilesional region, however, were epidermal rectification (Grenz zone), associated with the presence of disorganized perineural granulomas in the dermis, rich in xanthomatous histiocytes containing numerous bacilli, confirming the involvement by *Mycobacterium leprae* (Fig. 4). So the hypochromic spot suspected for leprosy was, in reality, an area of ​​the skin less affected by the disease on histopathology, when compared to the more severely affected area.

**Fig. 4:**
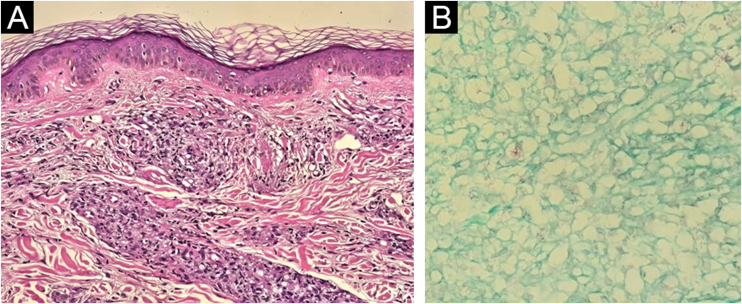
Histopathology of the region adjacent to the hypochromic spot seen in Fig. 2, Fig. 3: on the left, the presence of a Grenz zone and poorly organised granulomatous infiltrate, containing many xanthomatous histiocytes (Hematoxylin & eosin, ×100); on the right, presence of intact bacilli organized in clusters (Fite-Faraco, ×40).

Bacilloscopy was investigated, and the detected bacilloscopic index was 4.4. Once the diagnosis of borderline lepromatous L was confirmed, treatment with multibacillary multidrug therapy (MB-MDT) was initiated.

Leprosy is considered a “great disease imitator”, showing a wide variety of clinical manifestations, which can represent a diagnostic challenge in certain situations.^3^ There are reports of L mimicking diseases such as sarcoidosis, cutaneous lymphomas, Jessner's lymphocytic infiltrate and connective tissue diseases.^4^ The great variability of the clinical presentation reflects the hosts immunological spectrum in relation to *M. leprae* infection, which may show Th1 and Th2 pattern immune responses in polar clinical forms or both patterns in interpolar forms.^5^

Lymph node involvement in leprosy patients may occur as part of the visceral involvement in multibacillary patients. On histopathology, the reticuloendothelial system shows involvement and infiltration by *M. leprae* in a spectral pattern, similar to what occurs in the skin lesions. In lepromatous L, liver involvement occurs in 85% of cases and spleen involvement in approximately 41%, similar to what happened in the present case.^6^

There are reports of cases in which lymphomas simulate lepromatous L, or even the coexistence of both diseases.^4^, ^7^, ^8^ However, in the case described herein, the hypothesis of non-Hodgkin’s lymphoma was ruled out by the absence of atypical lymphocytosis in the blood count, as well as by the histopathology of the affected lymph nodes.

It is worth remembering that approximately one-third of patients diagnosed with multibacillary L may present with type II leprosy reaction (LR2) at the time of diagnosis, which makes it necessary to exclude the possibility of LR2 with lymph node infiltration in cases with lymph node enlargement.^9^ In the case described herein, the absence of pain, fever, neurological symptoms or lesions typical of erythema nodosum leprosum led to the suggestion of probable lymph node involvement in a patient with borderline lepromatous L. Additionally, there were no necrotic areas nor polymorphonuclear cell infiltration in the lymph nodes involved, as is usually observed in patients with lymph node leprosy reactions.^10^ In fact, four months after starting treatment, the patient developed erythematous nodules diffusely throughout the skin and neurological symptoms (significant pain on palpation of the ulnar nerves bilaterally and worsening of the palmoplantar esthesiometric examination), confirming the diagnosis of type 2 leprosy reaction following specific treatment.

In conclusion, it is essential that physicians closely monitor the possibility of involvement by leprosy bacilli in organs other than the skin and the peripheral nervous system. It is of utmost importance to emphasize the need to interrupt the chain of transmission, especially in multibacillary patients, such as the described case, so that, after diagnostic definition, they can be treated with multibacillary multidrug therapy.

## Financial support

None declared.

## Authors’ contributions

Victor Henrique Murback dos Reis: Design and planning of the study; collection of data, analysis and interpretation of data; drafting and editing of the manuscript or critical review of important intellectual content; approval of the final version of the manuscript.

Anna Carolina Miola: Design and planning of the study; drafting and editing of the manuscript or critical review of important intellectual content; intellectual participation in the propaedeutic and/or therapeutic conduct of the studied cases; critical review of the literature; approval of the final version of the manuscript.

Cesar Augusto Zago Ferreira: Design and planning of the study; collection of data, analysis and interpretation of data; drafting and editing of the manuscript or critical review of important intellectual content; approval of the final version of the manuscript.

Simone de Pádua Milagres: Design and planning of the study; drafting and editing of the manuscript or critical review of important intellectual content; intellectual participation in the propaedeutic and/or therapeutic conduct of the studied cases; critical review of the literature; approval of the final version of the manuscript.

Joel Carlos Lastória: Design and planning of the study; drafting and editing of the manuscript or critical review of important intellectual content; intellectual participation in the propaedeutic and/or therapeutic conduct of the studied cases; critical review of the literature; approval of the final version of the manuscript.

Juliano Vilaverde Schmitt: Design and planning of the study; drafting and editing of the manuscript or critical review of important intellectual content; intellectual participation in the propaedeutic and/or therapeutic conduct of the studied cases; critical review of the literature; approval of the final version of the manuscript.

## Conflicts of interest

None declared.
